# Improved forecasts of influenza-associated hospitalization rates with Google Search Trends

**DOI:** 10.1098/rsif.2019.0080

**Published:** 2019-06-12

**Authors:** Sasikiran Kandula, Sen Pei, Jeffrey Shaman

**Affiliations:** Department of Environmental Health Sciences, Columbia University, New York, NY 10032, USA

**Keywords:** influenza-like illness, hospitalization, search trends, forecasting, supervised learning

## Abstract

Reliable forecasts of influenza-associated hospitalizations during seasonal outbreaks can help health systems better prepare for patient surges. Within the USA, public health surveillance systems collect and distribute near real-time weekly hospitalization rates, a key observational metric that makes real-time forecast of this outcome possible. In this paper, we describe a method to forecast hospitalization rates using a population level transmission model in combination with a data assimilation technique. Using this method, we generated retrospective forecasts of hospitalization rates for five age groups and the overall population during five seasons in the USA and quantified forecast accuracy for both near-term and seasonal targets. Additionally, we describe methods to correct for under-reporting of hospitalization rates (backcast) and to estimate hospitalization rates from publicly available online search trends data (nowcast). Forecasts based on surveillance rates alone were reasonably accurate in predicting peak hospitalization rates (within ± 25% of the actual peak rate, three weeks before peak). The error in predicting rates one to four weeks ahead, remained constant for the duration of the seasons, even during periods of increased influenza incidence. An improvement in forecast quality across all age groups, seasons and targets was observed when backcasts and nowcasts supplemented surveillance data. These results suggest that the model-inference framework can provide reasonably accurate real-time forecasts of influenza hospitalizations; backcasts and nowcasts offer a way to improve system tolerance to observational errors.

## 1. Introduction

Seasonal influenza in the USA affects an estimated 9–35 million people annually with 140 000–710 000 resulting hospitalizations, a large proportion of which occur in the very young (less than 5 years of age) and the elderly (greater than 65 years) [1,2]. Surveillance systems that collect information on hospitalizations provide vital situation awareness to public health agencies. A useful complement to these surveillance systems are methods to forecast hospitalization rates in the near term—one to four weeks in the future—as well as trajectories of rates for the duration of outbreaks. Such forecasts can help with scheduling hospital staff, allocating hospital beds and ventilators, managing medical supplies and motivating the general public to get their flu shots or take other actions to reduce the spread of influenza.

Weekly estimates of influenza-associated hospitalization rates in the USA are publically available through the Centers for Disease Control and Prevention's (CDC) Influenza Hospitalization Network, FluSurv-NET (henceforth, FluSurv) [3]. As will be discussed in more detail in a later section, the hospitalization rates reported through FluSurv are often significant *underestimates* of actual hospitalization rates due to reporting delays of up to several weeks. Although these are corrected subsequently, forecasts made in real-time need to rely on these under-reported rates and hence can be erroneous.

We hypothesized that the quality of real-time forecasts could be improved by supplementing surveillance data with alternate data sources, specifically search trends data from the Google Extended Health Trends (GET) application programming interface (API). Estimating incidence of influenza-like illness (ILI) through query trends [4–7], Twitter feeds [8–10], access logs of informational sites and combinations of these sources has been studied quite extensively [11–14]; however, the focus has been largely directed at outpatient ILI incidence [15], which is an estimate of the proportion of patient visits to clinician offices attributable to ILI, and in a few cases, emergency department visits [16,17]. Compared to outpatient visits, hospitalizations result from more severe cases of influenza and estimating these cases poses a different challenge, and one arguably more critical during seasons when virulent strains circulate.

The advantages of using alternate rate estimates are twofold: (a) they provide more timely data, as GET, for instance, is updated daily, whereas FluSurv rates for a given week are first available with a one week delay and (b) as they are not governed by surveillance reporting protocols, the estimates are unlikely to be consistent underestimates, although errors and biases can certainly exist but are presumed to be smaller in magnitude than errors in FluSurv rates.

Despite these advantages, we are unaware of any prior work that used these data sources to estimate hospitalizations. In this paper, we first describe the application of a model-inference framework to forecast hospitalization rates based solely on FluSurv surveillance data. This forecast system was used to generate retrospective forecasts of hospitalization rates for five seasons and six age groups, and we report the accuracy of these forecasts in predicting six targets—seasonal peak weekly rate, the week during which the peak is predicted to occur, and rates one to four weeks in the future. We also generated variant retrospective forecasts based on observations from both FluSurv and GET, and report the improvement in the accuracy of the model-inference forecasts resulting from the inclusion of GET.

## 2. Data and methods

We first describe two sources for hospitalization rates: a national surveillance system for age-stratified laboratory-confirmed influenza hospitalizations, and an open API of online search trends for health-related terms. We then describe the two components of the forecast system, as well as its initialization and retrospective forecast generation process. We follow this by descriptions of a method to correct for under-reporting of hospitalization rates (backcast, also referred to as hindcast in numerical weather forecasting), a method to estimate hospitalization rates from search trends (nowcast), and end by describing alternative ways of incorporating these estimates into the forecasting system alongside surveillance data.

### 2.1. Influenza hospitalization network

FluSurv collects and disseminates data on laboratory-confirmed cases of influenza-associated hospitalizations in children and adults in the USA through population-based surveillance of a network of participating hospitals. The composition of the network has changed over time but has remained constant since the 2012/2013 influenza season. It currently includes about 250 participating hospitals from 70 counties in 13 states (CA, CO, CT, GA, MD, MI, MN, NM, NY, OH, OR, TN and UT) [18]. The distribution of the demographic (age, sex, race/ethnicity) and health indicators for the 27 million persons who are in the catchment area of this network is estimated to be similar to that of the national population; hence, it is standard practice to treat the hospitalization rates of the network as national rates [19].

Patients hospitalized during the influenza season (typically week 40 of a calendar year to week 18 of the following year) who have a documented positive influenza test are labelled as an influenza-associated hospitalization and are included in FluSurv. From these case counts, unadjusted rates are calculated using population estimates from the National Center for Health Statistics. Rates are publically available network-wide, at each individual state level, and disaggregated by patient age: 0–4 years, 5–17 years, 18–49 years, 50–65 years and 65+ years. An *Overall* age group with hospitalizations across all ages is also provided.

As the identification of influenza-associated hospitalizations requires laboratory confirmation, oversight by the clinicians in ordering a test, or in case of patients presenting with secondary complications from influenza, influenza no longer being detectable, can lead to under-reporting of hospitalization rates in real time. In addition, due to the significant time and effort required to link test results with admission databases and infection control logs, revisions are often made to estimates for multiple weeks after initial release.

**Figure 1.**
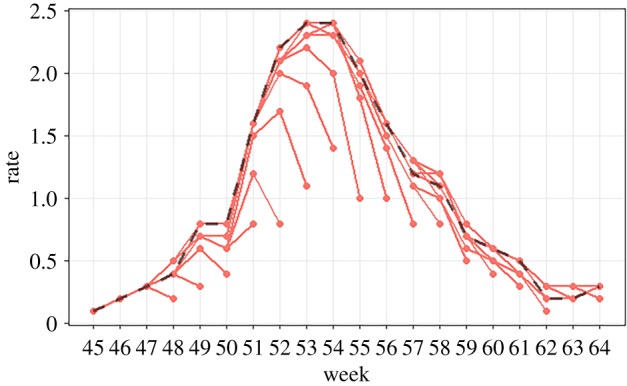
FluSurv rates for the *18–49 years* age group during 2013/2014 season with weekly revisions. The dashed black line shows the stabilized rates as determined at the end of the season. The individual trajectories, in red, show the rates available in real-time during different weeks of the season. For any given week, i.e. along any vertical line, the multiple data points show the different estimates of hospitalization rates for that week. For example, for week 53, the initial rate estimate was 1.1 (less than half the final rate of 2.4), which was revised up to 1.9 the following week, 2.2 the week after, etc. Generally, the initial estimates are almost always underestimates, but some of the intermediate rates can be overestimates. (Online version in colour.)

For example, figure 1 shows FluSurv rates for the *18–49 years* age group during the 2013/2014 season. The dashed black line shows the stabilized rates as determined at the end of the season and the individual trajectories in red show the rates available in real-time during different weeks of the season. The deviation of a trajectory from the stabilized rates is indicative of the magnitude of observational errors in real-time. In this case, revisions seem to occur for up to four weeks after the initial release, and are larger during periods of increased activity.

Retrospective forecasts made with stabilized rates can be expected to have higher forecast accuracy than those made in real-time when the available rates have large errors. To generate proper retrospective forecasts, as would have been generated in real time, hospitalization rates as they were first issued rather than the stabilized rates should be used. For the current analysis, we indeed used network-wide rates as available in real-time for the five seasons starting from the 2012/2013 season. This dataset was archived and available through the CDC's Epidemic Prediction Initiative [20].

### 2.2. Google Extended Health Trends API (GET)

The GET provides timeline data of the probability that a specified term is queried in the Google search engine. The trends data, available from January 2004, are calculated on a random sample of 10–15% of all searches, are updated daily and available at different geographical and temporal resolutions which can be specified through calls to the API.

In line with the available hospitalizations rates, we retrieved weekly trends data at the US national level beginning October 2012. Unlike hospitalization rates, trends data once released are not subsequently revised.

### 2.3. Model-inference framework

Forecasts were generated using a susceptible–infectious–recovered–susceptible (SIRS) compartmental model that has been iteratively optimized using a data assimilation algorithm—the ensemble adjusted Kalman filter (EAKF) [21].

#### 2.3.1. SIRS compartmental model

The SIRS model used here is a deterministic perfectly mixed, absolute humidity-driven model, which describes the propagation of influenza through a population. The model is defined as

2.1 $$\frac{dS}{dt}=\;\frac{N-S-I}{L}-\;\frac{\beta (t)IS}{N}-\;\alpha$$

and

2.2 $$\frac{dI}{dt}=\;\frac{\beta (t)IS}{N}-\;\frac{I}{D}+\alpha ,$$

where *S* is the number of susceptible individuals in the population, *t* is time, *N* is the population size, *I* is the number infectious, *N-S-I* is the number recovered, *β*(*t*) is the contact rate at time *t*, *L* is the average duration of immunity, *D* is the mean infectious period and *α* is the rate of travel-related import of influenza virus into the model domain (set to 0.1 infections per day).

The contact rate, *β*(*t*), is given by *β*(*t*) = *R*_0_(*t*)/*D*, where *R*_0_(*t*), the basic reproductive number, is the number of secondary infections an average infectious person would produce in a fully susceptible population at time *t*. Prior work [22,23] has demonstrated that specific humidity modulates influenza transmission and survival rates. Specifically, in laboratory experiments, specific humidity was found to explain 90% of the variability in influenza virus survival at 1 h and 50% of the variability in transmission among guinea pigs. Further analyses established that an exponential functional form best captures the relationship and is incorporated into estimation of *R*_0_(*t*) as:

2.3 $$R_{0}(t)=\;R_{0\min}+\;(R_{0\max}-\;R_{0\min})e^{-aq(t)},$$

where *R*_0 min_ and *R*_0max_ are the minimum and maximum daily basic reproductive number, respectively, *a* = 180 (estimated from the laboratory regression of influenza virus survival upon absolute humidity), and *q*(*t*) is the time-varying daily specific humidity. Daily averages were calculated from specific humidity observed over a 24-year period (1979–2002) as available from the National Land Data Assimilation System (NLDAS) project-2 dataset [24].

#### 2.3.2. Data assimilation

The SIRS model described above is defined by both observed (*I*, the number infectious/hospitalized) and unobserved quantities. Data assimilation is, broadly, the process by which observations are assimilated into a model structure so that the model state space provides a better representation of the actual system state. This is achieved by repeated correction of the observed and adjustment of the estimates of the unobserved. The EAKF is a sequential ensemble assimilation method used to estimate the conditional probability of observing a system state at time *t* given the series of observations through time *t*. It is sequential, as observations are iteratively assimilated through time, and an ensemble as the interrelationships between parameters/variables of the system are stored as multiple solutions of the system state, each of which is a possible realization of the state given the observations, rather than a single solution. Details of the method [21], and its application in conjunction with an SIRS model [25] are discussed elsewhere.

#### 2.3.3. Generating retrospective forecasts

The ensemble members in the model-inference system are initialized with parameter/variable values randomly selected from uniform distributions of pre-determined ranges. These ranges were identified by fitting the ensemble of simulations to known seasonal influenza outbreaks. See appendix 1 for the ranges used and an elaboration of the method. We report results with ensembles of 300 members, but forecast quality was found to be not very sensitive to ensemble size above 200.

To generate a forecast for an age group at a given week of a season, all available observations for the season up to the week of forecast are sequentially assimilated. That is, the ensemble of model simulations is initialized at week 40, which is considered to be the start of the influenza season, and integrated to the first observation. At this point, the simulations are halted and the EAKF is used to update the system state variables and parameters. The updated ensemble is then integrated to the next weekly observation and the adjustment process is repeated. Through this iterative assimilation of each weekly observation of estimated hospitalization rates, the EAKF filter adjusts the ensemble state space to better match the true system state. A forecast of hospitalization rates is generated after all observations up to a given week have been assimilated by integrating the posterior of each ensemble member through to the end of the season without further updating.

### 2.4. Backcasts

As described in §2.1, the hospitalization rates reported to FluSurv undergo substantial revisions during the course of the season as delayed data are reported. A majority are up-revisions, i.e. the initially released, partially observed rates tend to underestimate the final, fully observed rates. The availability of a data archive of these revisions [20] provides the opportunity to model these revisions and hence correct for reporting delays. We refer to an estimate of the fully observed rates from partially observed rates and historical revision data as a backcast.

Formally, let the partially observed FluSurv rate for age group *a* at week *w* per data released at week *v*, be denoted by $p_{w,l}^{a}$, where *a* ε [*0–4, 5–17, 18–49, 50–64, 65+, Overall*], lag *l = v* − *w*, and *v ≥ w*. The corresponding fully observed rate is denoted by $f_{w}^{a}$ and is determined at the end of a season. An age group specific random forest regression model is built with response variable $f_{w}^{a}$ and seven predictors {$\left\{l,p_{w,l}^{0-4},\cdots ,p_{w,l}^{\mathrm{Overall}}\right\}$}. Hence, each fully observed rate occurs in multiple instances of the training set, where the predictors in each instance are a snapshot of the partially observed rates of all age groups at a specific lag *l*. Let ${\overset{˘}{p}}_{w,l}^{a}$ denote the model's prediction—the backcast—of hospitalization rates for age group *a* during week *w,* per rates available *l* weeks from *w*.

To generate retrospective backcasts, separate models were trained for each season by excluding rates reported for all weeks of that season from the training set. The trained model is subsequently used to backcast for all weeks and lags of the season.

### 2.5. Nowcasts

Whereas backcasts are estimates for weeks where the FluSurv rates are available, nowcasts are GET based estimates of the hospitalization rates for weeks that have passed (when generated in real-time), but for which surveillance data are not available. Per the release schedule of FluSurv, at any given time in the season there is at least one week for which a nowcast can be generated.

The nowcast method used here is largely based on [26]. Briefly, we built regression models with the FluSurv reported rate as the response variable and search patterns of terms that are historically well correlated with FluSurv rates as explanatory variables. Separate models were built for the different age groups and the models were retrained at every week of a season.

#### 2.5.1. Feature identification

Google Correlate [4,27] was used to identify terms whose search frequency is highly correlated with FluSurv rates. This service, when provided with a candidate time series, returns a list of 100 query terms with the highest correlation to the candidate series. The query time series are weekly query fractions, whose numerator is the frequency with which a term/phrase was searched in the USA in a given time period and the denominator is the total number of queries in that period (to account for increase in search volume over time). As the number of potential query time series are very large, a two-step process is employed wherein an approximate distance between the candidate series and all series in the database is calculated in the first pass, and in the second pass, an exact distance is calculated only for the most promising series identified in the previous step [27].

By using FluSurv rates for the six age groups from the 2009/2010 season onwards as candidate trajectories, we identified a set of 280 terms to be used as features in regression models (the feature set is provided as supporting material to this manuscript). If *Q* denotes the feature set thus identified, *X*_1:*v*_, the feature matrix with the logit transformed trends data for weeks *1:v* is defined as

2.4 $$X_{1:v}\;\;=\;\;\left[\begin{array}{ccc} x_{1}^{1} & \ldots & x_{1}^{\left|Q\right|}\\ \vdots & \ddots & \vdots \\ x_{v}^{1} & \ldots & x_{v}^{\left|Q\right|} \end{array}\right]\;\;.$$

#### 2.5.2. Random forest regression model

Let $y_{1:w}^{a}$ denote the partially observed FluSurv rates for age group *a* through week *w*. Drawing on previous findings that simple autoregressive models for ILI provide reasonably good nowcast estimates [10,13], we fit an autoregressive integrated moving average (ARIMA) model [28,29] using observations through weeks *w* and predict (nowcast) ahead one week:

2.5 $${\tilde{y}}_{1:(w+1)}^{a}=\mathrm{ARIMA}(y_{1:w}^{a}).$$

The fit and prediction from the ARIMA model are added as an additional explanatory variable to the predictor matrix, yielding

2.6 $${\tilde{X}}_{1:(w+1)}^{a}=\;\;\left[\begin{array}{cc} X_{1:(w+1)} & \tilde{y}{{\;}_{1:(w+1)}^{a}}^{T} \end{array}\right].$$

Using ${\tilde{X}}_{1:w}^{a}$ as the predictor matrix and $y_{1:w}^{a}{\;}^{T}$ as the vector of responses, we train a random forest [30] model, ${\hat{f}}_{w}^{a}\;$, for age group *a* at week *w* as

2.7 $${\hat{y}}_{1:w}^{a}{\;}^{T}=\;{\hat{f}}_{w}^{a}\;\;({\tilde{X}}_{1:w}^{a}).$$

This model is then used with the predictor vector at week *w* + 1 to estimate rate at week *w* + 1*:*

2.8 $${\hat{y}}_{w+1}^{a}=\;\;{\hat{\;f}}_{w}^{a}\;\;({\tilde{X}}_{w+1}^{a}).$$

We only used rates per the most recent release, i.e. *l* = 0, to build nowcast models; in order to simplify the notation above, the lag term for FluSurv rates was dropped*.* R [31] packages *forecast* [32,33] and *randomForest* [34] were used for implementations of the ARIMA and random forest models, respectively.

### 2.6. Validation

Retrospective ensemble forecasts were generated using the optimized SIRS model for the 2012/2013 through 2016/2017 seasons for 20 weeks starting with MMWR [35] week 45 (roughly mid-November to late-March). Each age group was modelled separately with parameters initialized from uniform distributions as in appendix 1. The targets and comparison metrics used here were based on Epidemic Prediction Initiative's FluSight Collaborative guidelines [36]. Setting aside the 2012/2013 and 2013/2014 seasons for training, nowcasts were generated for the 2014/2015 season onwards.

#### 2.6.1. Variant retrospective forecasts

Let $y_{40:w}^{a}$ denote the FluSurv rates for age group *a*, up to week *w* during an influenza season (the season is generally considered to start at calendar week 40), ${\overset{˘}{p}}_{w,0}^{a}$ and ${\overset{˘}{p}}_{w,-1,1}^{a}$ the backcasts for the two most recent weeks for which FluSurv rates are available, and ${\hat{y}}_{w+1}^{a}$ the nowcast generated for week *w* + 1.

For each season and age group, we used five variant time series as observations for assimilation into the model-inference framework and hence had five variant retrospective forecasts:

— **L^F^**: Used FluSurv rates alone as observations i.e. $Y_{w}=\;y_{40:w}^{a}$
— **L^B^**: Replaced FluSurv rates of the two most recent weeks with their backcasts i.e. $Y_{w}\;\;=\;(y_{40:w-2}^{a},\;\;\;{\overset{˘}{p}}_{w,-1,1}^{a},\;\;\;{\overset{˘}{p}}_{w,0}^{a})$. This is based on a retrospective error analysis of the errors in backcasts which showed that only the backcasts at first two lags are on average more accurate than the corresponding partially observed rates.
— **L^N^**: Appended the nowcast estimate for week *w* + 1 to **L^B^**, i.e.$\;Y_{w}\;\;=\;(y_{40:w-2}^{a},\;\;\;{\overset{˘}{p}}_{w,-1,1}^{a},\;\;\;{\overset{˘}{p}}_{w,0}^{a},{\hat{y}}_{w+1}^{a})$. Note that the response variable for the nowcast model used to estimate ${\hat{y}}_{w+1}^{a}$ was same as *Y_w_* of **L^B^** and not **L^F^**.
— **L^Nk^**: Replaced FluSurv rates for the most recent *k* weeks with the corresponding fits from the nowcast model and appended the nowcast for week *w* + 1, i.e. $Y_{w}=\;(y_{40:(w-k)}^{a},{\hat{y}}_{\left(w-k+1):(w+1\right)}^{a})$. Here, we report results for *k* = {1, 2}.

See the electronic supplementary material, figure S1 for a schematic of the variant forecasts. The difference in forecast quality between **L^F^** and **L^B^** measures the value of backcasts in correcting under-reporting of FluSurv rates, the difference between **L^B^** and **L^N^** shows the added benefit of GET nowcasts, the difference between **L^B^** and **L^Nk^** informs whether nowcasts fits for past weeks improve over backcasts, and lastly the relative quality of **L^Nk^** forecasts helps identity the optimum window size for replacement. A **L^F^ - L^N^** comparison is appropriate for assessing the combined benefit of backcasts *and* nowcasts.

#### 2.6.2. Historical expectance as baseline

In the absence of forecasting models, estimates based on historical expectance—hospitalizations during a candidate season are hypothesized to be similar to hospitalizations in previous seasons—provide a reasonable baseline approach. In this study, as hospitalization data are available for relatively few seasons, we used a leave-one-out approach, i.e. observations from all available seasons, excluding the candidate season, were used to generate expectance based point and probabilistic forecasts for the candidate season. For each candidate season, the expectance point forecast of a target was the mean of the observed ground truth for that target in the remaining seasons. To obtain probabilistic forecasts, we used a Gaussian kernel density with a Sheather–Jones bandwidth [37]. This is similar to the baseline method used in the Epidemic Prediction Initiative's FluSight challenges [36].

#### 2.6.3. Targets

Two seasonal targets and four near-term forecasts are of interest and are defined as:

— Peak rate, the maximum weekly hospitalization rate observed during the season.
— Peak week, the MMWR week during which the maximum weekly hospitalization rate was observed.
— One- to four-week ahead forecasts, the estimates of hospitalization rates one to four weeks from the week of forecast. For example, for forecasts generated at week 50, the one- to four-week ahead forecasts are estimated rates for weeks 51–54.

It is necessary to note that the uncertainty of peak rate and peak week forecasts, unlike that of one- to four-week ahead forecasts, diminishes once the peak has occurred as the model posteriors tend to fit the observed rates quite well. The ground truth for all targets, age groups and seasons, was established using the fully observed rates (rounded to one significant digit) as available at the end of MMWR week 17 of the 2016/2017 season (electronic supplementary material, figure S2).

#### 2.6.4. Metrics

The model-inference framework was used to generate both probabilistic and point forecasts of the targets of interest. A probabilistic forecast is a set of probabilities assigned to the possible outcomes of the target. Given an ensemble trained with all observations available through week *w* for age group *a*, the probability assigned by the ensemble for one of the possible outcomes of a seasonal target, is the proportion of ensemble members predicting that particular outcome. For one- to four-week ahead targets, we define normal distributions on the mean and standard deviation of the individual ensemble member forecasts and the probability of observing any possible outcomes of the target is estimated from these distributions.

For peak week, the possible outcomes are MMWR week 40 through MMWR week 20. For the rate targets (peak rate and one- to four-week ahead rates), the possible outcomes are intervals of size 0.1 from 0 to 13, i.e. [0, 0.1), [0.1, 0.2)… [12.9, 13), [13,100]. As the 65+ age group usually has higher hospitalization rates, the upper bound of all rate targets for this age group was extended to 60.

Per Epidemic Prediction Initiative's FluSight Collaborative guidelines, the evaluation of a probabilistic forecast was based on the log score, $\ln\left(\sum_{i\;\in T_{g}^{a}}\;p_{i}\right)$, where $T_{g}^{a}$ is the set of acceptable outcomes of target *g* for age group *a* and *p_i_* is the probability assigned to outcome *i*. For peak week, an acceptable error margin of ±1 week was used, i.e. the acceptable outcomes were the exact peak week and the two weeks immediately adjacent to it. For the rate targets, an error margin of ±10% of the observed rate was used. For example, if the fully observed peak rate is 6 per 100 000, [5.4, 5.5) through [6.6, 6.7) were considered true outcomes. A perfect forecast (the sum of probabilities assigned to acceptable outcomes is 1) yields a log score of 0. A missed forecast (when none of the acceptable outcomes have a non-zero probability), is assigned a score of −10 in order to avoid a score of ln(0) = −∞).^1^

By contrast, a point forecast is the actual forecast value without the prediction intervals. For the model-inference ensemble, the mean of the ensemble trajectories is used to calculate point forecasts. For peak week, the point forecast is the week when the ensemble's mean trajectory peaks and the rate targets are the rates predicted by the ensemble mean, rounded to one significant digit. As measures of point forecast accuracy, we used absolute proportional error, i.e. the absolute error as a proportion of the fully observed rate. As weeks are ordinal, proportional error is inappropriate for peak week and in this case simple absolute error was used.

#### 2.6.5. Example

**Figure 2.**
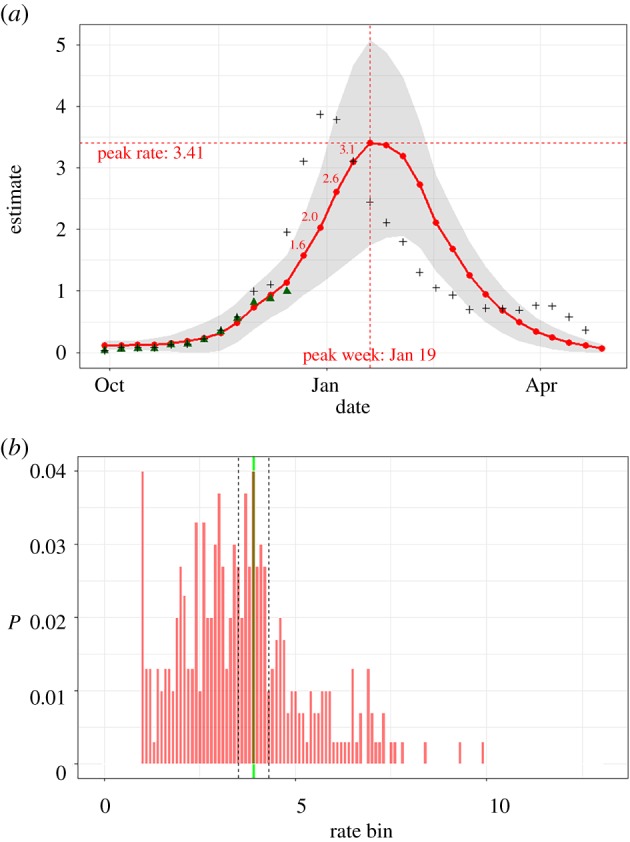
(*a*) Mean trajectory for week 51 of *Overall* age group during 2013/2014 season with predicted point forecasts for the six targets; green triangular points show observations (through week 51) used for data assimilation and the ‘+’ marks show the final observations (i.e. ground truth); the shaded region shows the ensemble spread (mean ± s.d.); (*b*) corresponding probabilistic forecast for peak rate along with the fully observed peak rate (green vertical line) and the valid bin range (black dashed vertical lines). (Online version in colour.)

Figure 2*a* shows an example forecast for the *Overall* age group during the 2013/2014 season. The forecasts were made using all observations (shown as green triangular points) available through week 51. The shaded region shows the ensemble spread (mean ± s.d.). The mean trajectory of the ensemble predicts the peak rate to be 3.4, five weeks in the future. Probabilistic forecast for one of the targets, peak rate, is shown in figure 2*b*.

The ground truth for peak rate for this season/age group, established at the end of season was 3.9. Hence the error in point forecast is |3.9–3.4|/3.9 = 0.1. The valid bin range for the probabilistic forecast is demarcated by vertical dashed lines and includes bins [3.5, 3.6) to [4.2, 4.3). The cumulative probability assigned to these eight bins is 0.16 and the log score is −1.83.

## 3. Results

A graphical examination of the ensemble forecast trajectories showed that the forecasts have no large anomalies. The distributions are reasonably narrow and tend to be centred on the true outcome in a majority of cases. See appendix 2 for an example.

**Figure 3.**
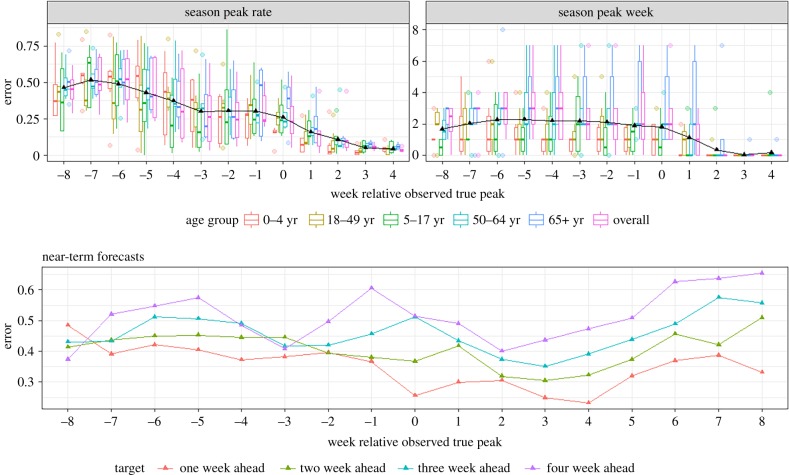
Mean absolute and mean absolute proportional errors of **L**^**F**^ forecasts for the six targets, across five seasons. (Online version in colour.)

Figure 3 shows the mean absolute proportional error of the peak rate and the mean absolute error of the peak week for the **L^F^** point forecasts (forecasts made with FluSurv rates alone). At eight weeks before the true peak, the mean error in peak rate was half the actual peak rate, decreased to a quarter of the peak rate at three weeks and was significantly lower after the peak has occurred. By contrast, no clear trend in peak week error was noted, possibly because of the consistently large errors in predicting the *65+ year* age group. However, the models were able to correct after the peak has passed and the error decreased considerably.

Figure 3 also shows the corresponding errors in near-term forecasts. As is to be expected the errors increase with increasing time horizon, i.e. for a given relative week, the one-week ahead errors are smaller than the two-week errors, which are in turn less than three-week errors, etc. It was reassuring to note that during periods of increased influenza activity (±3 weeks of peak) there were no corresponding increases in near-term forecast errors.

**Figure 4.**
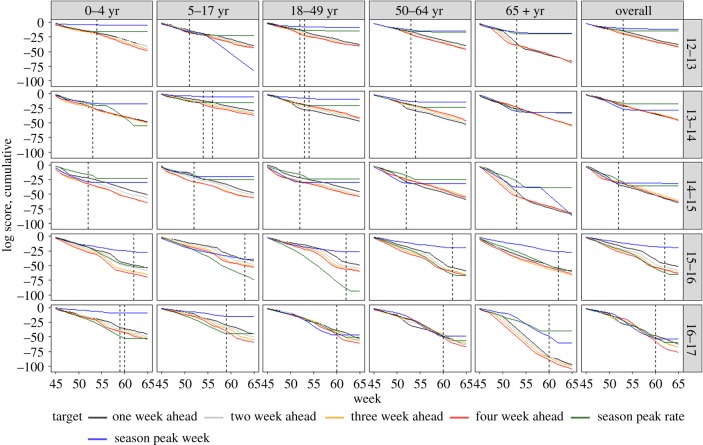
Cumulative log scores for **L**^**F**^ by season and age group. The dashed black line shows the peak week(s) for the season, age group combination. (Online version in colour.)

Figure 4 shows the rolling sum of log scores of the probabilistic forecasts of **L^F^** for each season and age group. Overall, peak week appears to have the lowest log score among the different targets but since the margins used for the rate targets and peak week are different, the scores are not directly comparable. Similar to what was observed with the point forecasts, the peak week predictions are accurate after the peak has occurred and the cumulative score line flattens.

A similar pattern is also seen for peak rate; however, in a few seasons and age groups the forecast misses the true outcome, and no levelling of the score is observed. As the margins used are proportional to the observed peak rate, when the peak rate is small the margins are quite narrow, and if there are revisions post hoc that shift the peak rate substantially, the probabilistic forecasts happen to miss the valid ranges. The decrease in forecast quality of the near-term forecasts with increasing forecast horizon that was observed for the point forecasts also holds true for the probabilistic forecasts. Together, these results from point and probabilistic forecasts using **L^F^** suggest that the model-inference framework is reasonably robust.

Table 1 shows the mean log scores and errors for all variant retrospective forecasts disaggregated by season, target and age group. Focusing on log scores, overall the scores of **L^B^** are higher than **L^F^**, demonstrating a clear benefit of replacing recent FluSurv rates with backcasts. With the inclusion of nowcasts (**L^N^**), scores improve further and are quite consistently the best among the variants examined. From scores of **L^N1^** and **L^N2^**, no discernable advantage from replacing backcasts with nowcasts is apparent.

**Table 1.** Mean errors and log scores of the variant retrospective forecasts by season, target and age. Lowest error and best score in each row is in bold. ‘ALL’ shows mean scores across all targets/groups for 14–15 to 16–17 seasons.

		scores						errors					
		*HistE*	L^F^	L^B^	L^N^	L^N1^	L^N2^	*HistE*	L^F^	L^B^	L^**N**^	**L**^**N1**^	**L**^**N2**^
ALL		−2.74	−2.62	−2.36	**−2.17**	−2.21	−2.26	—	—	—	—	—	—
season	12–13	−2.45	−1.80	**−1.79**	—	—	—	0.53	0.36	**0.32**	—	—	—
	13–14	−2.61	−1.82	**−1.79**	—	—	—	0.71	0.37	0.39	—	—	—
	14–15	−2.87	−2.46	−2.28	**−2.24**	−2.27	−2.25	0.47	0.49	0.45	0.44	0.43	**0.42**
	15–16	−3.10	−2.62	−2.34	**−2.20**	−2.29	−2.41	2.86	0.91	0.90	**0.68**	0.70	0.78
	16–17	−2.69	−2.78	−2.45	−2.07	**−2.06**	−2.13	.47	0.42	0.34	**0.29**	**0.29**	0.31
target	peak week	−1.93	**−1.65**	−1.74	−1.73	−1.79	−1.84	4.61	2.06	1.91	**1.9**	1.96	1.99
	peak rate	−2.82	−2.35	**−1.87**	**−1.87**	−1.88	−2.02	0.31	0.34	0.28	0.28	**0.27**	0.28
	one-week ahead	−3.14	−2.73	−2.33	**−2.13**	−2.25	−2.34	1.51	0.51	0.45	**0.41**	0.43	0.47
	two-week ahead	−3.16	−2.89	−2.57	**−1.64**	−1.71	−1.76	1.49	0.60	0.56	**0.45**	0.46	0.51
	three-week ahead	−3.14	−2.99	**−2.74**	−2.78	−2.78	−2.79	1.47	0.71	0.69	**0.55**	0.59	0.60
	four-week ahead	−3.13	−3.10	−2.90	−2.87	**−2.82**	−2.83	1.40	0.82	0.80	**0.65**	0.68	0.69
age group	0–4 years	−2.68	−2.33	−2.15	**−2.00**	−2.09	−2.12	1.41	0.58	0.57	**0.51**	0.54	0.59
	5–17 years	−2.90	−2.20	−2.06	**−1.96**	−1.97	−2.03	0.74	0.45	0.44	**0.42**	0.39	0.40
	18–49 years	−2.70	−2.44	−2.15	**−2.04**	−2.07	−2.13	1.04	0.51	0.47	0.43	**0.42**	0.45
	50–64 years	−2.86	−2.61	−2.21	**−2.06**	−2.14	−2.23	1.25	0.62	0.58	**0.48**	0.49	0.52
	≥ 65 years	−3.47	−3.44	−3.35	−2.89	**−2.84**	−2.87	1.61	0.81	0.75	**0.55**	0.56	0.56
	overall	−2.72	−2.70	−2.23	**−2.08**	−2.12	−2.20	1.34	0.58	0.51	**0.41**	**0.41**	0.45

When disaggregated by target, the peak rate scores are higher than the near-term forecast scores, which tend to decrease with increasing forecast horizons consistently for all variants. This is to be expected as errors in peak rate are largely limited to the part of the season preceding the peak, whereas the errors in near-term forecasts occur throughout the season. As electronic supplementary material, table S1 shows, when scores are limited to weeks preceding the peak, the one- and two-week ahead scores are indeed better than those of peak rate.

The superior performance of **L^N^** holds across all age groups. For each variant, the scores of all age groups are approximately equal with the exception of older adults (*65+ years*) where the highest hospitalization rates are usually observed. A more detailed analysis of this group showed that in a sizeable number of cases, the forecasts completely missed the true rates resulting in large penalty scores (−6.9, as described in §2.6.4).

**Figure 5.**
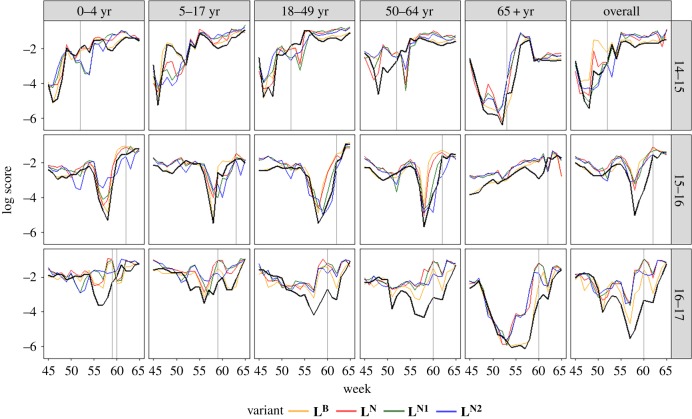
Mean log score across all targets for **L**^**B**^, **L**^**N**^ and **L**^**Nk**^ by age group and season. Corresponding scores for **L**^**F**^ are in black. The vertical grey line shows the peak week. (Online version in colour.)

Figure 5 shows the trends in log scores by age group within respective seasons, and we see that the advantage of the different variants over **L^F^** is not limited to any specific phase of the season. Periods of increased hospitalization rates (weeks immediately preceding the peak) are also weeks with the lowest scores.

Very similar results are observed with errors in point forecasts and here, too, **L^N^** quite consistently had the lowest errors by each disaggregation criteria (table 1). This insensitivity of the relative accuracy of the variant forecasts to the evaluation criteria used adds confidence that both the backcasts and nowcasts are useful for improving forecast accuracy.

With the exception of **L^F^** in a single instance, all variant methods presented here outperformed historical expectance based forecasts by both measures and across all disaggregation criteria examined (table 1, *HistE*).

## 4. Discussion

In this paper, we presented a method to forecast hospitalization rates, a method to accommodate for delayed reporting and a method to generate alternate estimates of hospitalizations from public data. The forecasting framework used here is similar to one being used to forecast outpatient ILI visits in the USA and the results presented here show that this approach is portable to hospitalizations. The improvement in forecast quality through the inclusion of backcasts and nowcasts shows that their respective advantages are perhaps additive and that they can, at least to some extent, compensate for the idiosyncrasies of this data stream.

During the last two influenza seasons, we have provided weekly real-time forecasts of hospitalization rates, generated with the models described here, to the CDC coordinated FluSight initiative. Assessments of the utility of these forecasts for public health planning, and the effect of their quality on decision-making are pertinent and need to be pursued. Furthermore, although the forecasts presented here are limited to US national-level forecasts, FluSurv rates are available at the state level for 13 states. It would be interesting to evaluate the utility of the methods described here in generating state-level forecasts as they, being more finely resolved, are likely to provide more directly actionable information to state departments of health and hospital networks than the national forecasts. An examination of the FluSurv rates at the state level showed that the issue of the initial under-reporting of rates was more pronounced and hence the forecasts would be more reliant on backcasts and nowcasts. However, as the nowcasts use FluSurv rates as the response variable, they are not immune to under-reporting, and the resulting nowcasts and forecasts may be of lower quality than the national estimates. This would potentially present a trade-off—more finely resolved, less accurate forecasts vis-à-vis coarser but more accurate forecasts—that is best resolved through operational comparison of these forecasts and input from public health officials.

In generating estimates for a season, the training sets of the different models excluded data from that season and used all other available seasons' data. This resulted in models for a season being trained on data from later seasons which would not have been possible if the estimates were being generated in real-time. More accurate simulation of real-time settings would have limited the size of the training set and potentially degraded backcast/forecast quality. Under the assumption that the seasons are independent of each other, and considering that models for future seasons would have at least six seasons' training data, we believe the out-of-sample approach used here is appropriate for comparing variant model forms.

Similarly, the initial parameter ranges for the inference system were identified using observations from seasons that were themselves being retrospectively forecast. This decision was made to address the paucity of historical data but could potentially have led to overfitting. In retrospect, alternate ways of identifying the initial ranges—for instance, a leave-one-out approach where the range to be used during a season is identified by exclusion of that season's outbreak—would have been more sound. However, we have no reason to believe that this choice can disproportionately affect one of the variant forecasts more than the others, thus the relative accuracy of these variants as reported will probably hold. We generated real-time forecasts for the 2017/2018 season using these same parameter priors and the log scores for this new season were found to be in line with the scores of retrospective forecasts presented here. Overfitting should have resulted in less accurate forecasts, suggesting that perhaps this was not the case.

We would also like to note that the compartmental model used in this study is quite simplistic and likely misspecified; for example, it does not explicitly model influenza infections that do not result in hospitalizations nor does it account for the age structure of the population. As discussed in earlier sections, a majority of the hospitalizations occur in two age groups and the transmission of influenza in an age group is influenced by factors and agents external to the population in the group. Exploration of more complex model forms that can capture a wider range of influenza infection severities and transmission across age groups is necessary and may yield more accurate forecasts, although the development of such model forecasts may require additional data sources for use in assimilation.

With respect to nowcasts, it has been previously reported that search trend-based proxy estimates of ILI from Google Flu Trends (GFT) have certain limitations, specifically issues related to transparency/reproducibility and potential for large errors in estimates [38–40]. Through the disclosure of the feature set of the nowcast model used here and access information for the underlying data (see the electronic supplementary material), we have attempted to address the concerns about openness [38]. While we have not reported stand-alone nowcast errors of hospitalization rates, our results show that the inclusion of these estimates in the forecast system (L^N*^ variants) improves forecast quality.

A related observation from table 1 is the lower score of one-week ahead forecasts for the L^N*^ variants than their two-week ahead forecasts, which is counter to the expectation of a degradation in forecast quality with increasing horizon. As the one-week ahead estimates for these models are nowcasts, and the two- to four-week ahead forecasts are forecasts from the mechanistic models, this discrepancy in scores is explainable, but it does suggest that the nowcasts can be substantially improved. Additionally, GET is not stratified by age and the query fraction matrix retrieved from GET are identical for all age groups. Hence, the nowcast models trained for different age groups differed only by the autoregressive feature of the predictor matrix and public data sources with real-time age stratified data, if available, may further improve the nowcasts and consequently the forecasts.

The choice of random forests for the backcasts and nowcasts is motivated by the suitability of the method for diverse domains, and by our familiarity with these models from prior work; however, we have yet to compare their performance with competing regression models in the context of hospitalizations. Exploration of alternative models is necessary to alleviate known bias problems of random forests—a tendency to over/underestimate extreme instances that are not represented in the training set—which could degrade the quality of backcasts and nowcasts during atypical seasons of unusually high hospitalization rates.

Finally, the probabilistic forecast scoring rules presented in this study are based on rules being used to compare multi-group forecasts. In addition to the 10% margins for the rate targets reported here, we used two alternate schemes—a margin of 5% relative to the true outcome and a fixed margin of five adjacent bins—and the findings were, for the most part, unchanged (results not presented).

## Supplementary Material

Supplementary information

## Supplementary Material

R script to analyze data and generate tables/plots used in the manuscript

## Supplementary Material

Forecast errors across all age group, seasons, weeks and targets

## Supplementary Material

Comprehensive RData archive of probabilistic forecasts

## Supplementary Material

Comprehensive RData archive of point forecasts

## Supplementary Material

Forecasts and metrics for baseline method

## Supplementary Material

List of terms used as features in the nowcast regression model

## Supplementary Material

RData archive of probabilistic nowcasts

## Supplementary Material

RData archive of probabilistic nowcasts

## Supplementary Material

R script of utility functions that can be used to generate nowcasts. Needs data from Google Extended Trends API.

## Supplementary Material

Log scores for probabilistic forecasts across all age group, seasons, weeks and targets

## Supplementary Material

Ground truth used to compute errors/scores for forecasts

## Endnote

1 We, however, post-process the forecast such that all outcome bins have at least *p* = 0.001, thus avoiding a ln(0) scenario. In effect, the lowest possible score for a forecast is −6.9.

## Data accessibility

US hospitalization rates data can be accessed at the FluView Hospitalization website [3]. A versioned archive of the rates which was used in the manuscript should be requested from the coordinators of EPI Prediction Initiative's FluSight [18] or accessed through the Delphi Research Group's Epidata API [41]. Similarly, access to Google's Extended Search Trends API has to be approved by Google. A list the search terms used as features to the nowcast model and an R script to generate nowcasts are provided as the electronic supplementary material. Implementation of the model-inference framework was published previously [42] and can be accessed here. An archive of nowcast estimates, retrospective point and probabilistic forecasts, accuracy measures calculated from the forecasts, and R code to reproduce tables/plots of the manuscript are also provided as electronic supplementary material.
