# Supplementary material for: Improved forecasts of influenza-associated hospitalization rates with Google Search Trends
Source: J R Soc Interface. 2019 Jun 12;16(155):20190080. doi: 10.1098/rsif.2019.0080 (PMC6597779; doi:10.1098/rsif.2019.0080)
Supplement: Supplementary information [file rsif20190080supp1.docx]

Improved Forecasts of Influenza Hospitalization Rates with Google Search Trends:

Supplementary information

Sasikiran Kandula, Sen Pei, Jeffrey Shaman

Department of Environmental Health Sciences, Columbia University, New York, NY, USA. 10032.

Corresponding author:

Sasikiran Kandula

Department of Environmental Health Sciences
722 West 168th Street
Allan Rosenfield Building, Room 1104A
New York, NY 10032

Email: [sk3542@cumc.columbia.edu](mailto:sk3542@cumc.columbia.edu)

APPENDIX 1

Initialization ranges for model parameters by age group identified from historical outbreaks. For each historical outbreak, we estimated the parameters ($\boldsymbol{R}_{\boldsymbol{0}\boldsymbol{max}}$, $\boldsymbol{R}_{\boldsymbol{0}\boldsymbol{min}}$, $\boldsymbol{D}$, and $\boldsymbol{L}$) using an iterative filtering algorithm Maximum Likelihood via Iterated Filtering (MIF) (1), where to achieve maximum likelihood, the distributions of parameters represented by an ensemble of possible system states are iteratively optimized. The estimated parameters were plugged back into the SIRS model, and through an exhaustive search on the grid of $\boldsymbol{S}_{\boldsymbol{0}}\boldsymbol{-}\boldsymbol{I}_{\boldsymbol{0}}$ plane, values for the susceptible ($\boldsymbol{S}_{\boldsymbol{0}}$) and infected ($\boldsymbol{I}_{\boldsymbol{0}}$) variables that best fit the epidemic curves were identified. The ranges for both parameters and variables were determined from the 95% credible intervals of corresponding distributions and are shown below:

| Age group | **S*1E6** | **I** | **R_0max_** | **R_0min_** | **L** | **D** |
| --- | --- | --- | --- | --- | --- | --- |
| 0 – 4 years | [.62, .72] | [1, 100] | [2.46, 3.05] | [1.03, 1.17] | [1875, 2514] | [3.80, 5.39] |
| 5-17 years | [.61, .71] | [1, 100] | [2.41, 3.11] | [1.02, 1.19] | [1912, 2563] | [3.25, 5.52] |
| 18-49 years | [.62, .71] | [1, 100] | [2.48, 3.12] | [1.03, 1.15] | [1917, 2519] | [3.54, 5.33] |
| 50-64 years | [.61, .71] | [1, 100] | [2.47, 3.21] | [1.03, 1.15] | [1904, 2514] | [3.11, 5.37] |
| ≥ 65 years | [.59, .69] | [1, 100] | [2.43, 3.33] | [1.02, 1.12] | [1950, 2473] | [2.86, 4.99] |
| Overall | [.62, .72] | [1, 100] | [2.50, 3.22] | [1.02, 1.13] | [1894, 2471] | [3.43, 5.47] |

APPENDIX 2

**Example L^F^ forecast for *Overall* age group during weeks 48 of 2013 through week 11 of 2014.** Each sub-panel shows the forecast made during a week with the cyan trajectory showing the posterior fit and the red trajectory the forecast. The prediction intervals (mean ± std. dev) are denoted by the shaded regions. The black triangles show the observations used and the solid black line the stabilized observations as determined at the end of the 2016/17 season. Note that the real-time rates do not match the stabilized rates (for example, weeks 52 and 53).


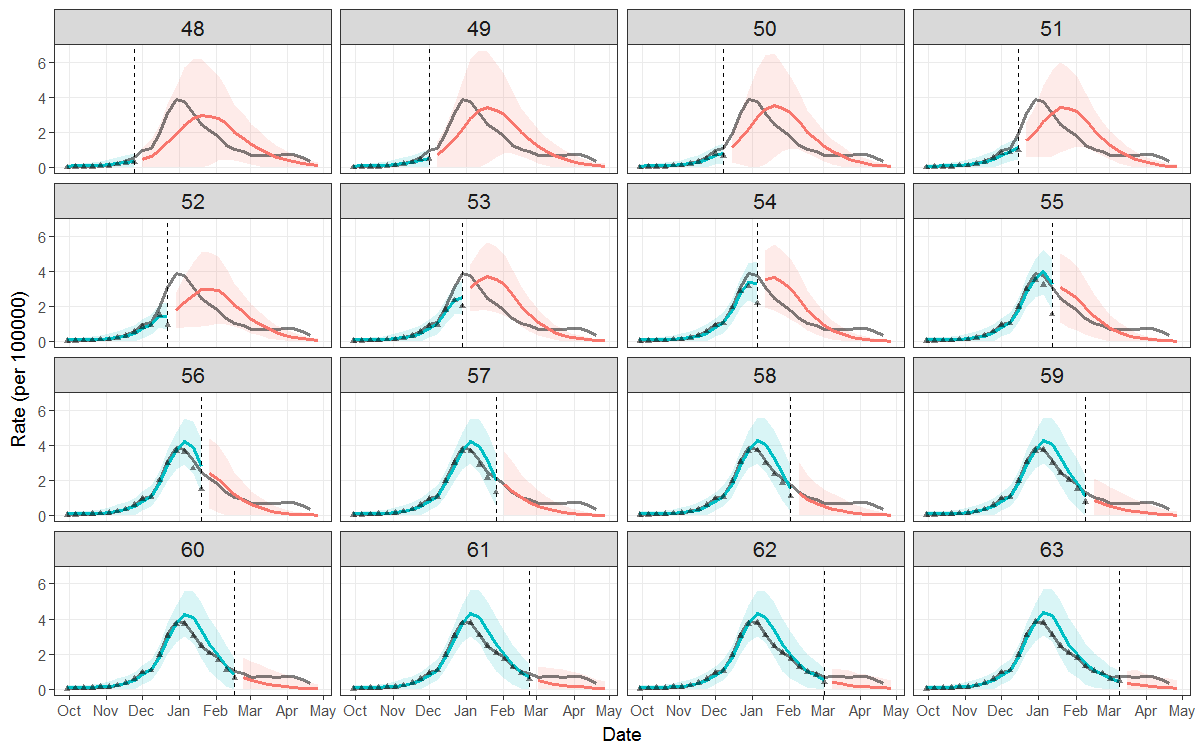


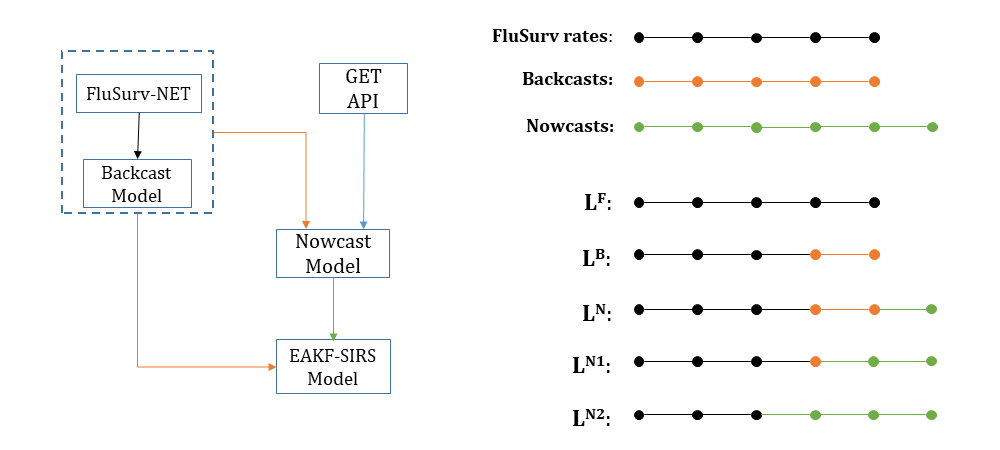


b)

a)

**Figure S1**. a) Schematic diagram showing flow of data within the forecast pipeline; b) representation of the observations used in each variant forecast. FluSurv-NET rates (in black), backcasts (in orange) and nowcasts (in green) are available to the model-assimilation framework. L^F-^ L^N2^ forecasts are generated using FluSurv-NET rates as observations or by different substitutions to these rates, as shown.


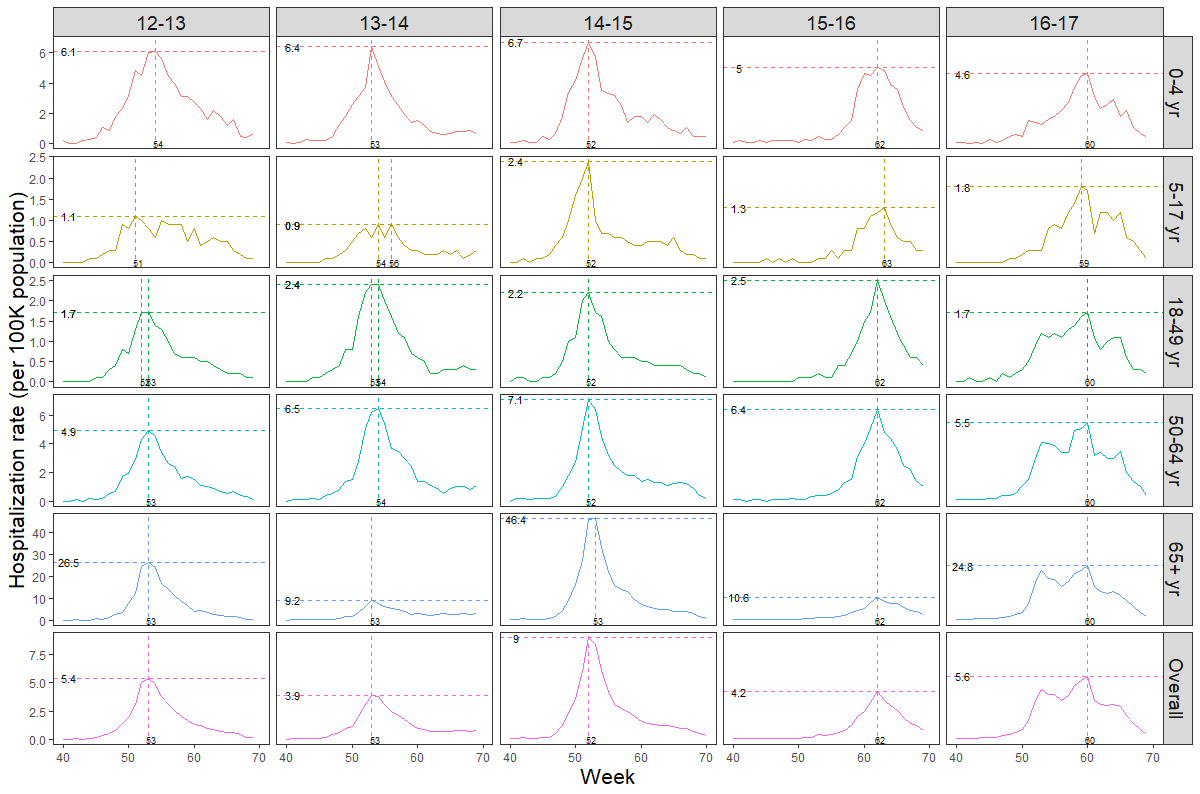


**Figure S2**. Plot of FluSurv hospitalizations rates for the 2012/13 through 2016/17 seasons, by age group. The vertical and horizontal lines indicate the week of season peak and peak rate respectively. In cases where peak rate, rounded to one significant digit, was reported for multiple weeks, peak week is not unique and this is indicated by multiple vertical lines in the subpanel.


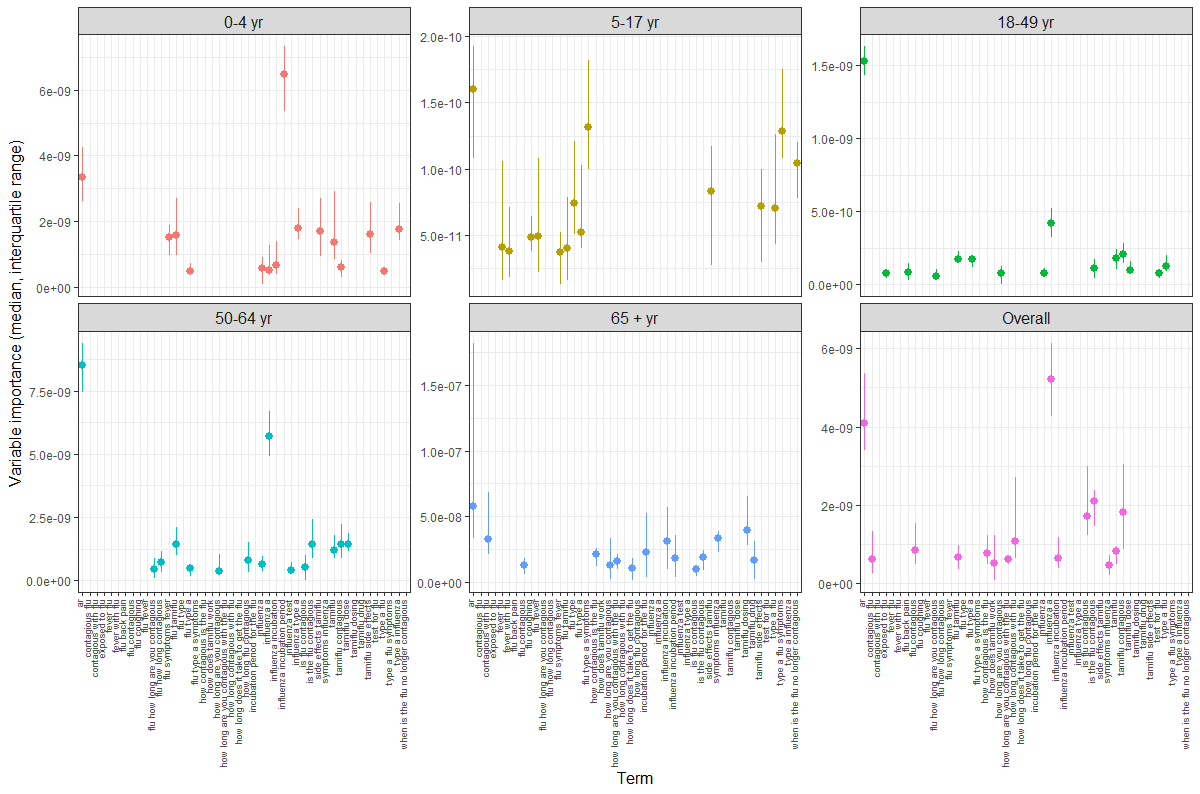


**Figure S3**. For each week in the 2016/17 season, random forest regression models were built for each age group and the variable importance estimated (broadly, change in mean squared error if the term were not used in the model). The plot shows the median and interquartile ranges of the variable importance of the top 15 terms (highest median) in each age group. The autoregressive term (*ar*) was found to be important in all age-specific models and there is noticeable variability both in terms of the top variables and their relative importance.

|  | L^F^ | L^B^ | L^N^ | L^N1^ | L^N2^ |
| --- | --- | --- | --- | --- | --- |
| Peak rate | -3.13 | -2.61 | -2.60 | -2.59 | -2.65 |
| 1-wk ahead | -2.96 | -2.43 | -2.30 | -2.41 | -2.48 |
| 2-wk ahead | -3.21 | -2.77 | -1.87 | -1.98 | -2.04 |
| 3-wk ahead | -3.35 | -3.02 | -3.11 | -3.11 | -3.13 |
| 4-wk ahead | -3.48 | -3.25 | -3.25 | -3.19 | -3.20 |

Table S1. Mean log score of the intensity targets of the variant retrospective forecasts, limited to weeks preceding the observed peak in each season and location. Lowest error and best score in each row is in bold.

REFERENCES

**[1]** Ionides EL, Bretó C, King A. Inference for nonlinear dynamical systems. Proceedings of the National Academy of Sciences. 2006;103(49):18438-43.
